# Overcoming apoptotic resistance afforded by Bcl-2 in lymphoid tumor cells: a critical role for dexamethasone

**DOI:** 10.1038/s41420-022-01285-x

**Published:** 2022-12-20

**Authors:** Carl D. Bortner, Robert H. Oakley, John A. Cidlowski

**Affiliations:** Signal Transduction Laboratory, Department of Health and Human Services, National Institute of Environmental Health Sciences (NIH), Research Triangle Park, Durham, NC 27709 USA

**Keywords:** Cell death, Cell signalling

## Abstract

Bcl-2 is an anti-apoptotic protein that promotes cell survival and resistance to cell death. Predictably, Bcl-2 as well as other anti-apoptotic Bcl-2 family members have been found to be overexpressed in a variety of human cancers. Approaches to overcome apoptotic resistance afforded by Bcl-2 in cells include anti-sense oligonucleotides, drugs that inhibit Bcl-2 function, and BH3 mimics have not been universally effective; thus, the need to understand the underlying mechanism of this resistance is vital. Glucocorticoids are stress hormones that act through their cognate receptors to control the transcription of numerous target genes, and in turn regulate a diverse array of biological processes. Synthetic glucocorticoids, such as dexamethasone, are prescribed in many chemotherapy protocols for neoplasms of lymphoid origin based on their ability to inhibit lymphocyte proliferation and promote apoptosis. However, lymphoid cells expressing Bcl-2 are resistant to glucocorticoid-induced cell death. We observed both pro- and anti-apoptotic characteristics in lymphoid cells expressing Bcl-2 following glucocorticoid treatment. These cells exhibited a profound change in their intracellular ionic composition, but a limited apoptotic ion flux and the absence of cell death. Provocatively, mimicking the loss of intracellular potassium using a low dose of a microbial toxin that acts as a potassium ionophore in combination with dexamethasone overcame the resistance afforded by Bcl-2 and killed the cells. Extending our study using other potassium ionophores revealed that direct depolarization of the mitochondria membrane potential coupled with prior treatment with glucocorticoids is the key mechanism for activating the cell death program and bypassing the resistance afforded by Bcl-2 in lymphoid cells. Finally, we show that the duration of dexamethasone pre-treatment is critical for regulating distinct genes and signaling pathways that sensitize the cells to die.

## Introduction

Glucocorticoids are primary stress hormones that function throughout the body to regulate a diverse array of physiological systems. These steroids act through their cognate receptors, regulate the transcription of numerous target genes that control/regulate various biological processes. Glucocorticoids have a profound impact on the immune system and are widely used as anti-inflammatory and immunosuppressive agents for many inflammatory and autoimmune diseases. This class of steroids are also well known to play a major role in cell death, notably in inducing apoptosis in lymphocytes [1, 2]. The regulation of apoptosis occurs through a balance of pro- and anti-apoptotic proteins, many belonging to the Bcl-2 family [3–5]. In many cell types, aberrant apoptotic activity is prevented by the expression of pro-survival (or anti-apoptotic) members of the Bcl-2 family, including Bcl-2, Bcl-xL, Bcl-w, Mcl-1, Bcl-B, and Bfl-1. Bcl-2 and other anti-apoptotic family members have been found to be over-expressed in a variety of cancers [6].

Overcoming apoptosis resistance afforded by the expression of Bcl-2 in cancer cells has been an area of intense research [7]. While various organic molecules and peptides have been developed, a class of drugs that mimic the pro-apoptotic BH3 domain-only proteins have been shown to be most effective in competitively binding with high affinity to the BH3 binding groove of anti-apoptotic Bcl-2 family members [8]. This action releases the pro-apoptotic molecules held in check by anti-apoptotic Bcl-2 proteins and induces cell death [9–11]. A BH3 mimetic ABT-737 was shown to bind Bcl-2 and Bcl-xL with high affinity killing primary cancer cells [9, 12, 13]. However, as Bcl-xL was found to be essential for the survival of mature platelets [14, 15], the effectiveness of ABT-737 was limited. While the translational success from preclinical studies to the clinic have been limited [5, 16], compounds such as Venetoclax that antagonize the anti-apoptotic function of Bcl-2 has been shown to be successful and approved for clinical use [17]. The success of these drugs has validated Bcl-2 as a therapeutic target for cancer, however further studies into overcoming the resistance to cell death afforded by the expression of anti-apoptotic proteins in cancer cells is needed to develop additional therapeutics.

S49.1 cells are an immature mouse lymphoma cell line that is highly sensitive to killing by glucocorticoids [18, 19]. Expression of Bcl-2 in S49.1 cells (S49 Bcl-2 cells) results in a marked resistance to cell death upon treatment with the synthetic glucocorticoid dexamethasone [20]. The nature of protection against glucocorticoid-induced cell death in S49 Bcl-2 cells has been an active area of investigation over the past 25 years [21–24]. We examined S49 Bcl-2 cells to determine if any components of apoptosis were present following dexamethasone treatment and observed the presence of both pro- and anti-apoptotic characteristics in the absence of cell death. We hypothesized that exploiting one or more of the established glucocorticoid-associated apoptotic characteristics may sensitize S49 Bcl-2 cells to undergo cell death. Here we show that the addition of non-lethal doses of specific microbial toxins which result in depolarization of the mitochondrial membrane potential induces cell death of S49 Bcl-2 cells pretreated for a requisite period of time with glucocorticoids.

## Results

### Bcl-2 prevents development of characteristics associated with glucocorticoid-induced apoptosis in S49 T cells

**Fig. 1:**
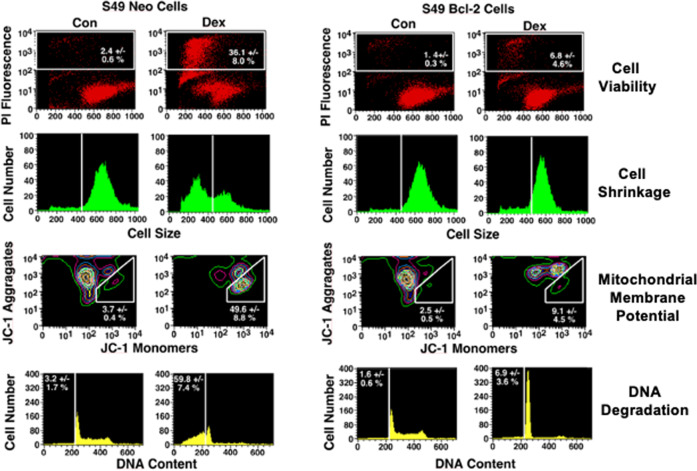
Bcl-2 prevents classical characteristics associated with dex-induced apoptosis in S49 T cells. S49 (Neo) cells treated with 2.5 × 10^−7^ M dex for 48 h resulted in the loss of membrane integrity as determined by the increase in the number of cells which were stained with the vital dye, propidium iodide (PI), an increase in the number of cells which had a decrease in their ability to scatter light in the forward direction, indicating a population of cells with decreased cell size, depolarization of the mitochondrial membrane potential (MMP), evident by the increase in JC-1 monomers and decrease in JC-1 aggregates, and the presence of a sub-diploid peak of DNA, indicative of degraded DNA. In contrast, S49 (Bcl-2) cells treated with dex under identical conditions did not result in any significant changes in membrane integrity or cell size. Interesting, mitochondrial membrane hyperpolarization was observed along with a block at the G1/S phase of the cell cycle. Data represent the mean (+/− SEM) of 3-5 independent experiments.

We examined S49 (Neo) and S49 (Bcl-2) cells for the occurrence of characteristics of apoptosis including loss of membrane integrity, cell size, changes in the mitochondrial membrane potential, and DNA integrity in response to dexamethasone (dex) by flow cytometry. Treatment of S49 (Neo) cells with dex for 48 h resulted in the loss of cell viability, an increase in the number of smaller or shrunken cells, a depolarized mitochondrial membrane potential (MMP), and the occurrence of a sub-diploid peak of DNA, indicative of degraded DNA (Fig. 1, left). Together these data indicate that the activation of cell death with dexamethasone in S49 (Neo) cells results in apoptosis. In contrast, S49 (Bcl-2) cells treated with dex under identical conditions did not result in any significant changes in membrane integrity or cell size, however a block at the G1/S phase of the cell cycle was observed (Fig. 1, right). Interestingly, S49 (Bcl-2) cells treated with dex resulted in a stark hyperpolarization of the MMP, as the number of cells that had JC-1 aggregates appeared increased with the absence of JC-1 monomers (Fig. 1, right). Thus, while the expression of Bcl-2 is widely believed to be a potent inhibitor of glucocorticoid-induced apoptosis in lymphoid cells, our data shows that dex treatment in cells expressing Bcl-2 does not result in a complete absence of glucocorticoid effects but shows a restricted response to glucocorticoid treatment. To confirm the presence of Bcl-2 in the S49 cells, we examined Bcl-2 expression by flow cytometry using an anti-human Bcl-2 antibody. Only the S49 (Bcl-2) cells showed the presence of Bcl-2 (Supplementary Fig. 1),

### S49 (Bcl-2) cells treated with dex results in hyperpolarization of both the mitochondrial and plasma membrane potentials

**Fig. 2:**
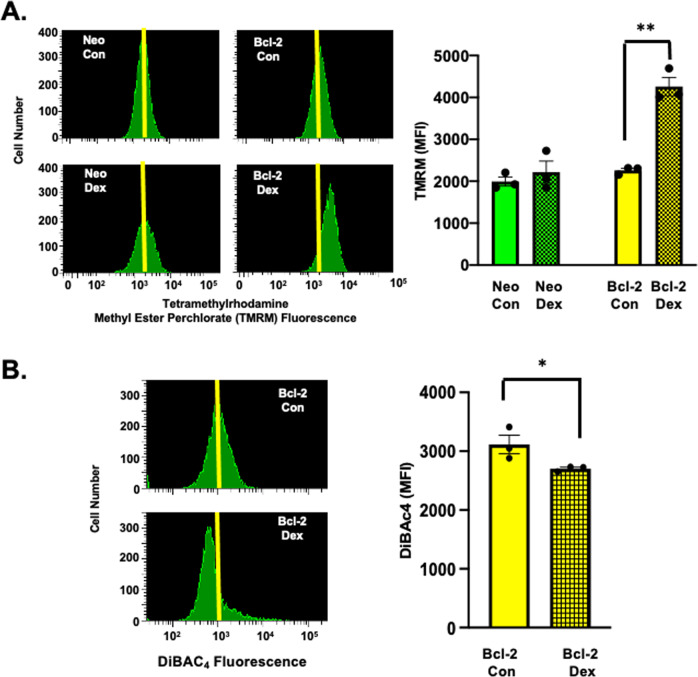
S49 (Bcl-2) cells treated with dex results in hyperpolarization of the mitochondrial and plasma membrane potential in the viable population of cells. **A** S49 (Neo) and S49 (Bcl-2) cells treated in the presence or absence of dex for 48 h were examined for changes in their mitochondrial membrane potential (MMP) using tetramethylrhodamine methyl ester perchlorate (TMRM; 50 nM), 30 min prior to flow cytometry analysis. The viable S49 (Neo) dex-treated cells showed little change in their mean fluorescent intensity (MFI) compared to control cells, however the coefficient of variation (dispersion of the distribution) appeared broader. In contrast, the viable S49 (Bcl-2) dex-treated cells showed a dramatic increase in TMRM fluorescence, suggesting a hyperpolarized MMP. **B** S49 (Bcl-2) cells treated in the presence or absence of dex for 48 h were examined for changes in their plasma membrane potential (PMP) using DiBAC_4_(3) (150 ng/ml final), 30 min prior to flow cytometry analysis. S49 (Bcl-2) cells treated with dex showed a decrease in DiBAC_4_(3) fluorescence indicating a significant hyperpolarization of the PMP, similar to what was observed for the MMP. Data represent the mean (+/− SEM) of 3 independent experiments. *p < 0.05; **p < 0.01.

The stark increase in JC-1 aggregates in the dex-treated S49 (Bcl-2) cells led us to further examine changes in the MMP. S49 (Neo) and S49 (Bcl-2) cells treated in the presence or absence of dex for 48 h were examined for changes in their MMP by flow cytometry using tetramethylrhodamine methyl ester (TMRM; Fig. 2A). The surviving S49 (Neo) dex-treated cells showed little change in their mean fluorescent intensity (MFI) compared to their corresponding control cells, however the coefficient of variation (dispersion of the distribution) appeared broader (Fig. 2A). In contrast, all the surviving S49 (Bcl-2) dex-treated cells showed a dramatic increase in TMRM fluorescence (Fig. 2A), suggesting hyperpolarization of the MMP, similar to that observed in the S49 (Bcl-2) cells stained with JC-1 (Fig. 1).

To evaluate if changes in the MMP were reflected in changes in the plasma membrane potential (PMP), we examined S49 (Bcl-2) cells for changes in the PMP by flow cytometry using the anionic membrane potential dye DiBAC_4_(3). This negatively charged dye is free to cross the plasma membrane and will increase in intensity upon cellular depolarization. S49 (Bcl-2) cells loaded with DiBAC_4_(3) and treated with dex for 48 h showed a decrease in DiBAC_4_(3) fluorescence compared to the control cells indicating a significant hyperpolarization of the PMP (Fig. 2B). This data suggests that the expression of Bcl-2 has a profound effect on the intracellular ionic composition of dex-treated cells resulting in the hyperpolarization of both the MMP and PMP that occurs in the absence of cell death.

### S49 (Bcl-2) cells treated with dex results in a high percentage of cells with increased reactive oxygen species (ROS)

**Fig. 3:**
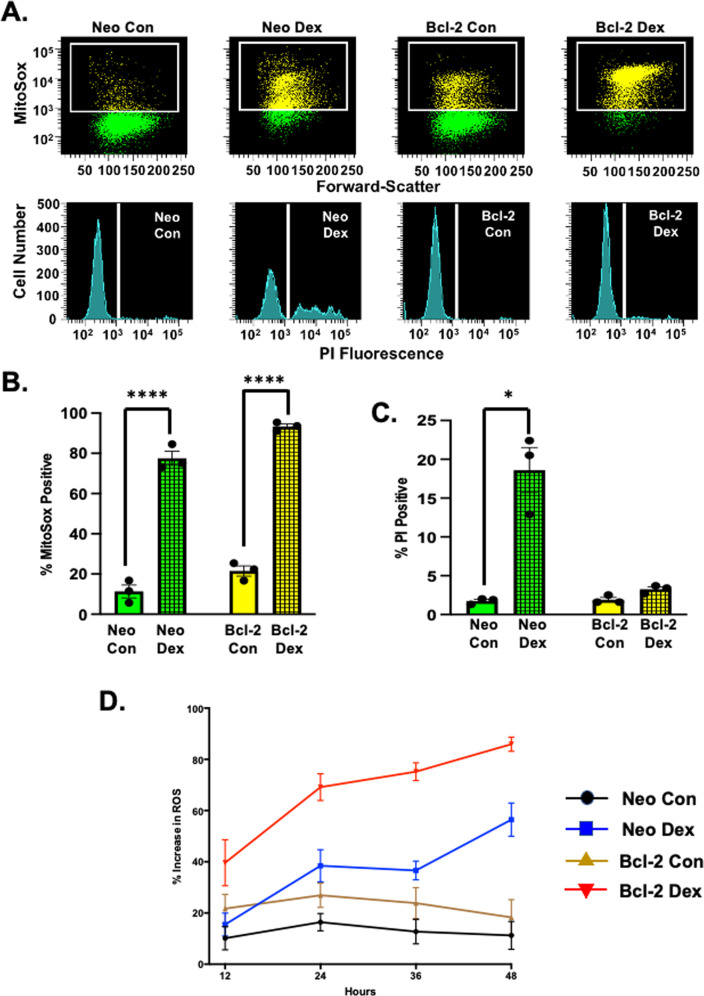
S49 (Bcl-2) cells treated with dex results in a high percentage of cells with increased reactive oxygen species (ROS). **A** S49 (Neo) and S49 (Bcl-2) cells treated in the presence or absence of 2.5 × 10^−7^ M dex for 48 h were examined for generation of reactive oxygen species (ROS) using MitoSox Red (5 uM, 30 min) by flow cytometric analysis. S49 (Neo) cells treated with dex showed an increase in ROS that resulted in cell death. Dex-treated S49 (Bcl-2) after 48 h resulted in a population of cells with a high level of ROS, without the loss of cell viability. **B** Quantitation of the change in ROS shown in **A**. Data represent the mean (+/− SEM) of 3 independent experiments. **C** Quantitation of the loss in membrane integrity shown in **A**. Data represent the mean (+/− SEM) of 3 independent experiments. **D** S49 (Neo) and S49 (Bcl-2) cells treated with 2.5 × 10^−7^ M dex were examined for ROS generation over time. Data represent the mean (+/− SEM) of 5 independent experiments. *p < 0.05; ****p < 0.0001.

To further evaluate the effect glucocorticoids have on S49 (Bcl-2) cells, we examined dex-treated S49 (Bcl-2) cells for additional cellular events and/or characteristics related to the cell death program. The generation of reactive oxygen species (ROS) is a common feature of cells undergoing cell death. S49 (Neo) cells treated dex for 48 h showed a population of cells with increased ROS, along with an increase in the number of dead cells (Fig. 3A–C). Interestingly, 48 h of dex-treatment in the S49 (Bcl-2) cells resulted in nearly 100% of the cells with a high level of ROS, in the absence of cell death (Fig. 3A–C).

Additionally, examining S49 (Neo) and S49 (Bcl-2) cells over time in the presence and absence of glucocorticoids showed the dex-treated S49 (Bcl-2) cells had a higher percent of cells with increased ROS, when compared to the dex-treated S49 (Neo) cells independent of time (Fig. 3D). This data suggests that glucocorticoids (dex) have a dramatic effect on cellular physiology over time in cells expressing Bcl-2; however, the accumulation of ROS alone is not concomitant with cell death.

### Bcl-2 blocks alterations in intracellular Na+ and K+ induced by dex in lymphoid cells that undergo apoptosis

**Fig. 4:**
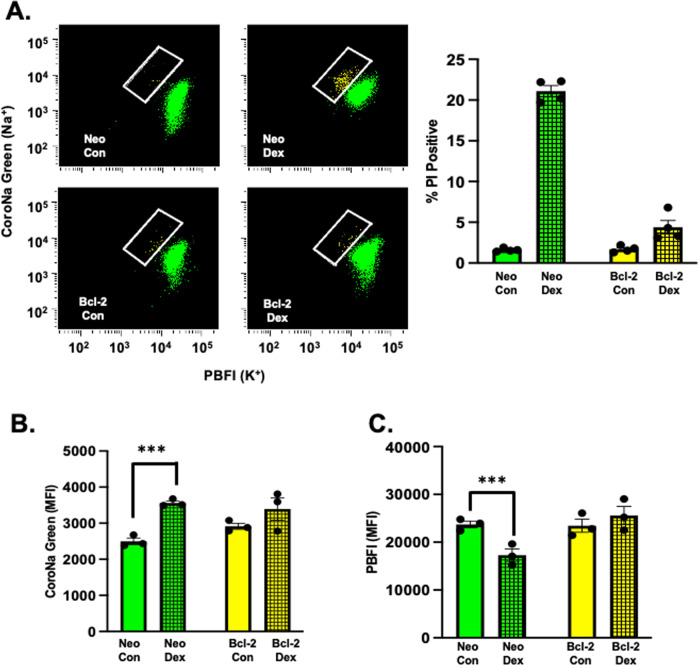
Absence of classical apoptotic intracellular ionic changes in S49 (Bcl-2) cells treated with dex. **A** S49 (Neo) and S49 (Bcl-2) cells treated with and without 2.5 × 10^−7^ M dex for 48 h were examined by flow cytometry for changes in intracellular sodium and potassium using CoroNa Green and PBFI-AM, respectively. Flow cytometric analysis of the viable dex-treated S49 (Neo) cells showed a cell population with increased intracellular sodium and decreased intracellular potassium, resulting in cell death. In contrast, dex-treated S49 (Bcl-2) cells did not show the occurrence of this population of cells, and did not die. Analysis of mean fluorescent intensity (MFI) for CoroNa Green **(B)** and PBFI **(C)** showed a significant increase in intracellular sodium coupled with a decrease in intracellular potassium only in the viable S49 (Neo) cells treated with dex. Data represent the mean (+/− SEM) of 3-4 independent experiments. ***p < 0.001.

Previous studies from our lab have shown that cells undergoing apoptosis have a unique ionic signature during an initial stage of cell volume loss that occurs prior to the loss of membrane integrity; an early loss of intracellular potassium coupled with a simultaneous increase in intracellular sodium [25]. We examined S49 (Neo) and S49 (Bcl-2) cells treated in the presence or absence of dex for 48 h for changes in their intracellular sodium and potassium content. As shown in Fig. 4A, S49 (Neo) cells treated with dex results in a viable population of cells with an increase in intracellular sodium coupled with an initial loss of intracellular potassium that occurs prior to cell death. Interestingly, this cell population is not observed in the dex-treated S49 (Bcl-2) cells (Fig. 4A). Analysis of the change in the mean fluorescent intensity (MFI) for each ion showed only the S49 (Neo) cells had a significant increase in intracellular sodium and loss of intracellular potassium (Fig. 4B, C, respectively). These changes in intracellular ions were absent in dex-treated S49 (Bcl-2) cells, though a trend towards an increase in intracellular sodium in the dex-treated S49 (Bcl-2) cells was observed (Fig. 4B). Additionally, we examined the cells for changes in intracellular calcium. However, as the response of S49 cells to dexamethasone is rather stochastic, a significant increase in intracellular calcium was not observed after 48 h of treatment (Supplementary Fig. 2).

### Addition of a very low dose of the potassium ionophore microbial toxin valinomycin to dex-treated S49 (Bcl-2) cells mimics the ion flux observed during apoptosis and kills the cells

**Fig. 5:**
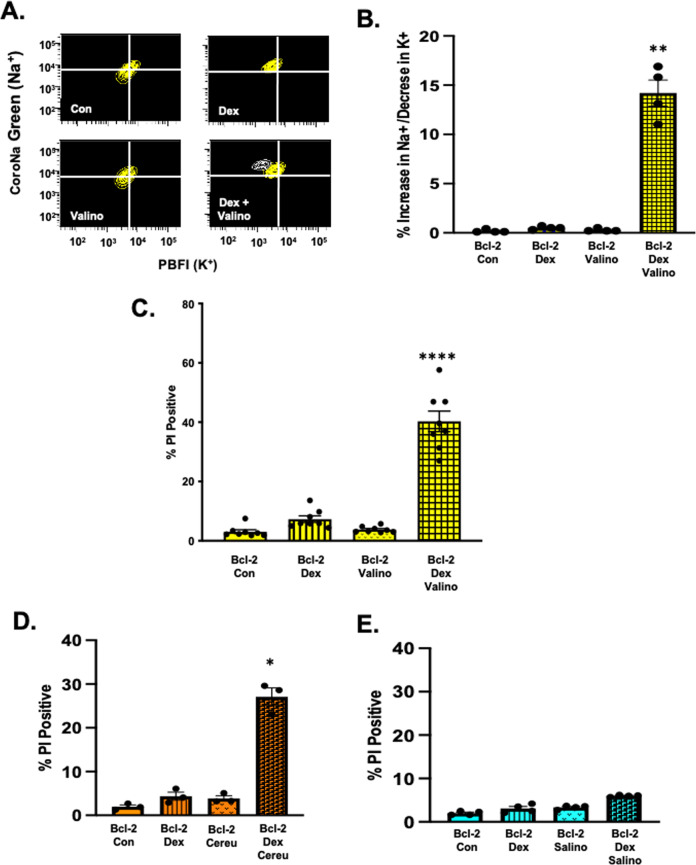
A low dose of the microbial toxin valinomycin mimics the ion flux observed during apoptosis and kills dex-treated S49 (Bcl-2) cells. **A** S49 (Bcl-2) cells treated with 2.5 × 10^−7^ M dex for 48 h where a low dose (25 nM) of valinomycin was added during the final 6 h were examined for changes in intracellular sodium and potassium using CoroNa Green and PBFI-AM, respectively. **B** Quantitation of the population of cells with increased sodium and decreased potassium shown in **A**. Data represent the mean (+/− SEM) of 4 independent experiments. **p < 0.01 **C** S49 (Neo) and S49 (Bcl-2) cells treated with 2.5 × 10^−7^ M dex for 48 h where a low dose (25 nM) of valinomycin was added during the final 6 h were examined for a loss of membrane integrity using PI and flow cytometry. Data represent the mean (+/− SEM) of 3-4 independent experiments, ****p < 0.0001. S49 (Bcl-2) cells were treated with 2.5 × 10^−7^ M dex for 48 h in the presence of absence of **D** 25 nM cereulide, or **E** 10 uM salinomycin during the final 6 h and examined for cell viability by flow cytometry using PI. Data represent the mean (+/− SEM) of 3-4 independent experiments, *p < 0.05.

The change in both the MMP and PMP coupled with observed changes in intracellular ions in dex-treated S49 (Bcl-2) cells suggests that the expression of this proto-oncogene limits the activation of the cell death process by affecting ion flux and the loss of intracellular potassium. Our previous studies have shown that the loss of intracellular potassium is a key event for the activation/activity of the apoptotic machinery [26–28]. Thus, we hypothesized that an imposed flux or loss of intracellular potassium from dex-treated cells expressing Bcl-2 may result in an intracellular ionic environment conducive for cell death. To test this hypothesis, we employed a very low concentration of the well-known microbial toxin valinomycin (a potassium ionophore) that allows potassium to efflux from the cell via its normal concentration gradient. The addition of 25 nM of valinomycin during the final 6 h of the 48 h dex treatment in S49 Bcl-2 cells resulted in an enhanced ion flux with a well-defined population of cells with increased sodium and decreased potassium (Fig. 5A, B), characteristic of cells undergoing apoptosis. Additionally, valinomycin treatment during the final 6 h of a 48 h dex treatment in S49 (Bcl-2) cells resulted in cell death, while cells treated with either dex or valinomycin alone remained resistant to death (Fig. 5C).

We expanded the scope of our study to examine other microbial toxins that act as potassium ionophores for their ability to overcome apoptotic resistance in cells expressing Bcl-2 treated with glucocorticoids. Cereulide, a toxin produced by *Bacillus cereus*, acts as an ionophore with high affinity to potassium cations, and salinomycin is an ionophorous antibiotic isolated from *Streptomyces* spp. shown at high concentrations to have K^+^ selectivity resulting in the rapid release of K^+^ from the mitochondria [29]. Additionally, salinomycin has been shown to effectively kill breast cancer stem cells at micromolar concentrations [30] and is thought to be an effective anticancer drug [31–35]. S49 (Bcl-2) cells were treated with dex for 48 h in the presence or absence of cereulide or salinomycin during the final 6 h of glucocorticoid treatment and examined for cell viability by flow cytometry. In the absence of dex, neither drug had any effect on cell viability. Interestingly, only cereulide, like valinomycin, when added during the final 6 h of a 48 h dex treatment resulted in cell death in the S49 (Bcl-2) cells (Fig. 5D). In marked contrast, salinomycin had no effect in killing lymphoid cells expressing Bcl-2 (Fig. 5E), even at a 400-fold greater concentration, suggesting that valinomycin and cereulide provide a unique cellular signal to kill glucocorticoid-primed Bcl-2 cells.

### Mitochondrial depolarization provides the key to kill dex-primed S49 (Bcl-2) cells

**Fig. 6:**
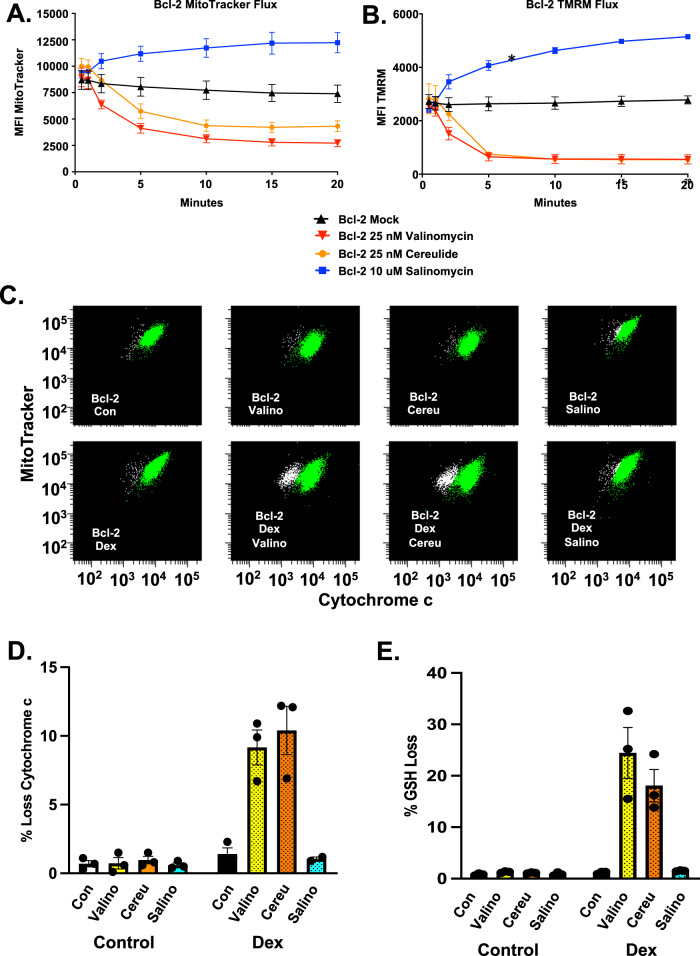
Microbial toxins that override apoptotic resistance in Bcl-2 expressing cells depolarize the mitochondrial membrane potential. S49 (Bcl-2) cells loaded with either 25 nM MitoTracker Deep Red (**A**) or 50 nM tetramethylrhodamine methyl ester perchlorate (TMRM; **B**) then treated with 25 nM valinomycin, 25 nM cereulide, or 10 uM salinomycin were examined for changes in their mitochondrial membrane potential (MMP) by flow cytometry. Data represent the mean (+/− SEM) of 3 independent experiments. S49 (Bcl-2) cells were treated with 2.5 × 10^−7^ M dex for 48 h in the presence or absence of 25 nM valinomycin, 25 nM cereulide, or 10 uM salinomycin during the final 6 h and examined for the loss of cytochrome c (**C**, **D**) and lose of glutathione (**E**). Data represent the mean (+/− SEM) of 3 independent experiments.

Earlier we showed that dex treatment resulted in a protective hyperpolarization of the MMP (Fig. 2). To investigate the unique cellular signal afforded by valinomycin and cereulide in killing glucocorticoid-primed Bcl-2 cells, we examined their effect on the MMP. We used two different indicators of MMP, both mark cells based on the proton electrochemical gradient potential of the MMP. S49 (Bcl-2) cells stained with either MitoTracker Deep Red or TMRM were examined over time in the absence or presence of the various drugs. Mock treated S49 (Bcl-2) cells loaded with MitoTracker Deep Red or TMRM did not show any significant change in MMP over a 20 min period (Fig. 6A, B, black line). S49 (Bcl-2) cells loaded with MitoTracker Deep Red or TMRM and treated with either valinomycin (red line) or cereulide (orange line), showed a rapid decrease in dye intensity that continued over 20 min, suggesting a prompt and sustained depolarization of the MMP (Fig. 6A, B). Interestingly, salinomycin (blue line) showed an increase in dye incorporation into the cells suggesting this drug does not depolarize but hyperpolarizes the MMP in S49 (Bcl-2) cells. These studies suggest that both valinomycin and cereulide have a pro-apoptotic effect (MMP depolarization) to kill Bcl-2 cells when primed by glucocorticoids.

Previous studies have suggested that MMP depolarization alone was not sufficient to overcome the resistance afforded by Bcl-2, and release of cytochrome c and/or depletion of cellular glutathione were also required [36]. Thus, we examined S49 (Bcl-2) cells under our experimental conditions for downstream effects of MMP depolarization, including release of cytochrome c and loss of glutathione. Only S49 (Bcl-2) cells exposed to dex for 48 h with the addition of either valinomycin or cereulide during the final 6 h showed a release of cytochrome c from the viable population of cells (Fig. 6C, D). Additionally, we also only observed the loss of glutathione (GSH) in these samples (Fig. 6E). S49 (Bcl-2) cells exposed to dex or dex plus salinomycin that did not result in the release of cytochrome c or GSH loss (Fig. 6C–E). Furthermore, in the absence of dex, no downstream effects of MMP depolarization were observed in response to these drugs alone. Therefore, the ability of valinomycin and cereulide to depolarize the MMP in cells primed by glucocorticoids provides the signal to trigger the apoptotic process, resulting in the loss of cytochrome c and GSH to ultimately override the protection afforded by Bcl-2 and kill the cells.

Interestingly, as a stochastic response of an increase in intracellular calcium was observed upon 48 h dex treatment in the S49 (Neo) cells undergoing apoptosis, the rapid activation of the cell death process upon valinomycin and cereulide addition in the dex treated S49 (Bcl-2) cells showed a more systemic response (Supplementary Fig. 3).

**Fig. 7:**
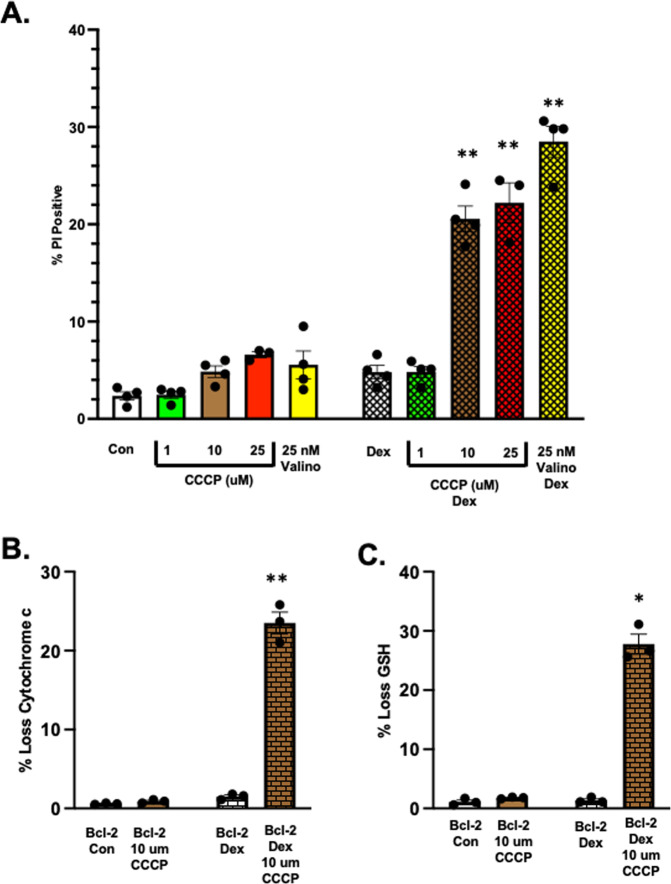
Mitochondrial depolarization provides the key to kill dex-primed S49 (Bcl-2) cells. **A** S49 (Bcl-2) cells treated with 2.5 × 10^−7^ M dex for 48 h in the presence of absence of 25 nM valinomycin, or various concentrations of CCCP during the final 6 h were examined for cell viability by flow cytometry. S49 (Bcl-2) cells treated with 2.5 × 10^−7^ M dex for 48 h in the presence of absence of 10 uM CCCP during the final 6 h were examined for the loss of cytochrome c (**B**) and lose of glutathione (**C**). Data represent the mean (+/− SEM) of 3 to 5 independent experiments *p < 0.05; **p < 0.01.

To directly test the hypothesis that mitochondrial depolarization is the critical event in overriding the resistance afforded by Bcl-2 in dex-primed cells, we analyzed the effect of carbonyl cyanide m-chlorophenylhydrazone (CCCP) a known mitochondrial uncoupling and membrane depolarization agent. Initially, we examined various concentrations of CCCP in the context of our model system. Whereas 1 uM CCCP had no effect, 10 uM was sufficient to trigger cell death in 48 h dex-primed S49 (Bcl-2) cells to a similar level as observed with valinomycin (Fig. 7A). Using this concentration of CCCP, we examined these cells for the loss of cytochrome c and glutathione. Similar to the observations with valinomycin or cerulide, 10 uM CCCP also resulted in the loss of both cytochrome c and glutathione (Fig. 7B, C), suggesting that drugs that depolarize the mitochondria can overcome the resistance afforded by Bcl-2 in cells pre-treated or primed with glucocorticoids.

### Duration of glucocorticoid treatment is critical and results in the regulation of unique genes and signaling pathways in S49 (Bcl-2) cells

**Fig. 8:**
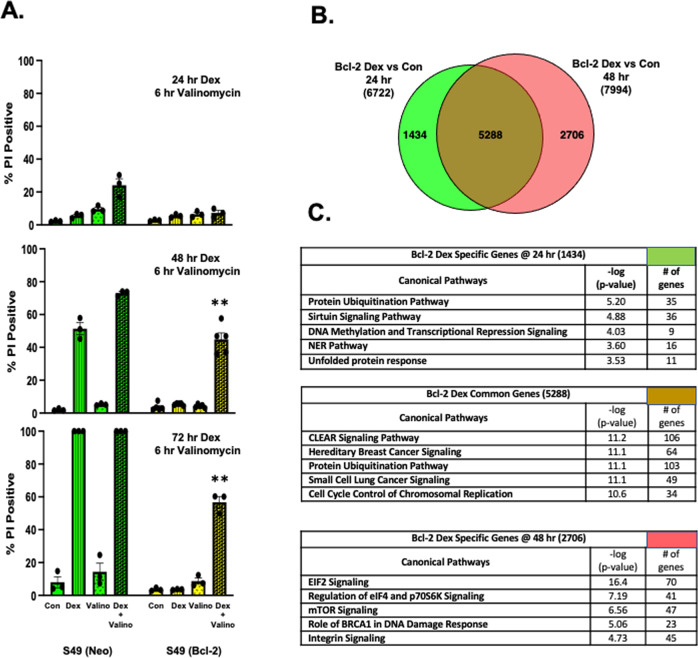
Analysis of cell death in S49 (Neo) and (Bcl-2) cells and gene expression profile of S49 (Bcl-2) cells treated with dexamethasone for 24 h or 48 h. **A** S49 (Neo) and S49 (Bcl-2) cells were examined in for changes in their membrane integrity after the addition of valinomycin during the final 6 h of a 24 h, 48 h, or 72 h dex treatment. Data represent the mean (+/− SEM) of 3-5 independent experiments. **B** Venn diagram analysis of dysregulated genes after 24 h dex-treatment versus dysregulated genes after 48 h dex-treatment in S49 (Bcl-2) cells. **C** Ingenuity pathway analysis (IPA) showing the top 5 canonical signaling pathways associated with 24 h dex-specific, common, and 48 h dex-specific genes in S49 (Bcl-2) cells.

The strategic role glucocorticoids play in overcoming the apoptotic resistance afforded by Bcl-2 was examined at different times prior to the addition of valinomycin. As shown in Fig. 8A, the addition of valinomycin during the final 6 h of a 24 h dex treatment had no effect in killing the S49 (Bcl-2) cells. However, this paradigm did enhance cell death in the S49 Neo cells. The addition of valinomycin during the final 6 h of a 48 h dex treatment resulted in killing of the S49 (Bcl-2) cells, and again enhanced the killing in the S49 (Neo) cells (Fig. 8A). The addition of valinomycin during the final 6 h of a 72 h dex treatment shows only a slight increase in the percent of dead S49 (Bcl-2) cells compared to the 48 h dex treatment (Fig. 8A). This data suggests that the duration of glucocorticoid treatment plays a critical role in priming Bcl-2 expressing cells to undergo cell death in the presence of valinomycin.

To determine how the glucocorticoid signaling profile is influenced by treatment time, we performed RNAseq on S49 (Bcl2) cells treated with and without dex for 24 h and 48 h. Overall, dex regulated a similar number of genes at 24 h and 48 h (6722 and 7994, respectively; Fig. 8B). Venn diagram analysis of these two gene sets revealed that most of the genes (5288) were commonly regulated by dex independent of the treatment time. However, 1434 genes were uniquely regulated by dex at 24 h, and a greater of genes, 2706, were uniquely regulated by dex at 48 h. Ingenuity pathway analysis (IPA) was performed to determine the canonical signaling pathways most strongly associated with these common and unique gene sets (Fig. 8C). The top 5 signaling pathways associated with the 2706 genes uniquely regulated by dex at 48 h included EIF2 signaling, Regulation of eIF4 and p70S6K signaling, mTOR signaling, and the Role of BRCA1 in DNA Damage Response. These pathways play critical roles in the regulation of protein synthesis, cell proliferation and survival, and DNA repair and are completely different not only from the pathways associated with the 1434 genes uniquely regulated by dex at 24 h, but also from the pathways associated with the 5288 commonly regulated genes. These data demonstrate that a 48 h dex treatment of S49 (Bcl2) cells leads to the regulation of unique gene sets and signaling pathways that can sensitize the cells to die upon mitochondrial depolarization.

## Discussion

The expression of Bcl-2 protects lymphoid cells from cell death induced by glucocorticoids. However glucocorticoid treatment in lymphoid cells expressing Bcl-2 develop several pro- and anti-apoptotic features in the absence of cell death. Dex-treated S49 (Bcl-2) cells results in hyperpolarization of both the mitochondrial and plasma membrane potentials, and a lack of early intracellular apoptotic ionic changes. In contrast, these cells also show an increase in mitochondrial-derived reactive oxygen species, a trait largely considered to be pro-apoptotic, however these changes in ROS alone were not associated with cell death. Our data suggest that glucocorticoid treatment has a profound effect on cells expressing Bcl-2 without killing them.

To further examine the consequence of these glucocorticoid-induced changes in lymphoid cells expressing Bcl-2, we administrated a low dose of the potassium ionophore valinomycin during the final 6 h of a 48-hour dex treatment. The combination glucocorticoid treatment plus the valinomycin in S49 (Bcl-2) cells overrides the protection afforded by Bcl-2 resulting in the activation of the cell death program. When extending our study to other potassium ionophores in combination with glucocorticoids, discovered that only drugs that depolarized the mitochondrial membrane potential triggered the apoptotic response and killed glucocorticoid-primed Bcl-2 cells.

Several key observations were noted in this study that are critical in inducing apoptosis in lymphoid cells expressing Bcl-2. First, an absolute requirement for the cells to be treated with glucocorticoids. In the absence of glucocorticoids, apoptotic agents alone are benign in their ability to kill cells. Second, the stimulus must have the ability to depolarize the mitochondrial membrane potential. Both valinomycin and cereulide immediately depolarize the mitochondrial membrane potential, whereas salinomycin hyperpolarized the MMP. The importance of a drugs ability to depolarization the mitochondrial membrane potential was confirmed in our studies using CCCP. Importantly, our data shows that MMP depolarization alone does not override the protection to cell death afforded by Bcl-2, but the glucocorticoid-sensitization of these cells towards cell death combined with a mitochondrial depolarization signal results in a rapid killing of the cells. A third key observation is the importance of the duration of glucocorticoid treatment to bring the cells to the threshold of cell death. As illustrated in this study, 24 h dex treatment did not provide sufficient time to prime the cells to die upon addition of a death stimulus. However, 48 h of dex treatment led to the regulation of unique gene sets and signaling pathways that sensitized the cells to die upon mitochondrial depolarization. Thus, glucocorticoids play a critical role in activating the apoptotic process by providing time-dependent gene changes that permit the rapid triggering of the cell death process. Interestingly, the observed significance of the EIF2-signaling pathway, essential for protein synthesis and a key adaptive survival response [37], also suggests that changes at the level of protein expression may play a critical role in the ability of dexamethasone to sensitize cells expressing Bcl-2 to die.

The initial protection afforded by Bcl-2 may in part be due to the dex-induced hyperpolarization of the MMP, an action opposite to the depolarization of the MMP shown here to be a key signal of the cell death mechanism. Salinomycin resulted in hyperpolarization of the MMP, thus possibly further protecting the cells from cell death. Both valinomycin and cereulide resulted in the immediate depolarization of the MMP, which was sustained over time. The drug-induced MMP depolarization coupled with dex-induced gene changes at 48 h has a direct impact in the subsequent release of pro-apoptotic factors from the mitochondria including cytochrome-c, which in turn results in the loss of glutathione, known to be involved in mechanism of cell death. Additionally, as BH3-only proteins are known to play an important role during dex-induced apoptosis [38, 39], our RNAseq analysis did not suggest any significant transcriptional change in pro-apoptotic proteins. However, it cannot be ruled out that glucocorticoid treatment may affect the delicate balance between pro- and anti-apoptotic proteins, including their non-apoptotic functions that controls the cells fate [40, 41].

In patients with relapsed or relapsed and refractory multiple myeloma, the combination of daratumumab, bortezomib, and dexamethasone resulted in significantly longer progression-free survival then if these drugs were used alone [42]. Additionally, the combination of glucocorticoids with inhibitors of Bcl-2 or MCL-1 such as venetoclax and bortezomib, respectively, has been shown to be a promising and safe treatment for relapsed/refractory multiple myeloma [43]. A current mechanism behind the effectiveness of this treatment suggests a dex-induced increase in the expression of both Bcl-2 and Bim, which alters the binding of Bim to the anti-apoptotic proteins [44]. While our study did not result in a significant increase in Bim expression, our data suggests that glucocorticoids may afford additional pro-apoptotic mechanisms permitting the depolarization of the mitochondrial membrane potential to activate the cell death process.

In conclusion, persistent glucocorticoid treatment positions S49 (Bcl-2) cells at the brink of cell death, where low doses of agents that depolarize the mitochondrial membrane potential initiates the cell death process, essentially pushing the cells over the edge. The resulting release of cytochrome c and loss of glutathione then completes the cell death process, effectively killing cells expressing the anti-apoptotic protein Bcl-2. A limitation of the current study is the use of mouse cells expressing Bcl-2; thus, it would be of great value to examine this effect in human cancer cells. However, as our study shows, therapeutic drug combinations that include glucocorticoids can promote specific/separate components of the cell death process resulting in a more effective induction of apoptosis in cells expressing Bcl-2.

## Material and methods

### Cell culture and chemicals

S49 (Neo) are S49.1 mouse lymphoma cells stably transfected with a recombinant amphitropic retrovirus carrying a G418 (Neomycin) antibiotic resistance gene [20]. S49 Bcl-2 cells were additionally transfected with the Bcl-2 proto-oncogene [20]. Both cell lines were cultured in RPMI-1640 medium containing 10% heat-inactivated fetal calf serum, 4.8 mM glutamine, 100 ug/ml streptomycin and 100 U/ml penicillin at 37 °C, 7% CO_2_ atmosphere. Dexamethasone was purchased through Steraloids (Wilton, NH). Valinomycin, cereulide, and salinomycin were purchased from Thermo Fisher Scientific (Invitrogen; Hillsboro, OR), Focus Biomolecules (Plymouth Meeting, PA), and EMD Millipore Corporation (Seattle, WA), respectively.

### Determination of Bcl-2 expression by flow cytometry

Expression of Bcl-2 was analyzed using a Fixation and Permeabilization Kit and a PE Mouse Anti-Human Bcl-2 Set (BD Biosciences, Cat. No. 556535) according to the manufacturer’s instructions.

### Determination of plasma membrane integrity, cell size, and DNA content by flow cytometry

Plasma membrane integrity was determined with the addition of propidium iodide (PI; Sigma) to a final concentration of 10 ug/ml added immediately prior to analysis. Changes in cell size and DNA content were determined by flow cytometry as described previously [45] using a Becton Dickinson FACSort equipped with Cell Quest software (BD Biosciences).

### Analysis of mitochondrial membrane potential

Changes in the mitochondrial membrane potential were measured by flow cytometry using JC-1 (Molecular Probes) as previously described [46]. Tetramethylrhodamine methyl ester perchlorate (TMRM; ThermoFisher) or MitoTracker Deep Red (Moleular Probes) were added to a final concentration of 200 nM during the final 30 min of incubation, using with Sytox Blue or PI as a vital dye, respectively. All samples were analyzed by flow cytometry using an LSRII (BD Biosciences) equipped with FACSDiVa software for acquisition and analysis.

### Determination of changes in plasma membrane potential

Thirty minutes prior to the point of examination, DiBAC_4_(3) (Invitrogen; Molecular Probes) was added to 1 ml of cells to a final concentration of 150 ng/ml and incubation was continued at 37 °C, 7% CO_2_ atmosphere. All samples were analyzed by flow cytometry using an LSRII (BD Biosciences) equipped with FACSDiVa software for acquisition and analysis.

### Flow cytometric analysis of ROS

Analysis of mitochondrial ROS was determined by adding MitoSox Red (5 uM final; Life Technologies) to each sample for 30 min at 37 °C, 7% CO_2_ atmosphere prior to examination using Sytox Blue as a vital dye. All samples were analyzed using a Becton Dickinson LSRII equipped with FACSDiVa software for acquisition and analysis. Only cells that did not lose their membrane integrity (Sytox Blue negative) were included in the analysis.

### Determination of intracellular potassium, sodium, and calcium

Analysis of intracellular sodium and potassium by flow cytometry was accomplished as described previously [25]. Briefly, 2 ul of 2.5 mM CoroNa Green-AM (Na^+^) or PBFI-AM (K^+^) (Molecular Probes) stock were added to 1 ml of cells for a final concentration of 5 uM 1 h prior to the time of examination. Intracellular calcium was determined by adding 4 ul of 1 mM Fluo-4 (Life Technologies) to 1 ml of cell for a final concentration of 4 uM 30 min prior to the time of examination. Incubation was continued at 37 °C, 7% CO_2_ atmosphere. Immediately prior to flow cytometric examination, propidium iodide (PI) was added to a final concentration of 10 ug/ml. All samples were analyzed by flow cytometry using an LSRII (BD Biosciences) equipped with FACSDiVa software. Only cells that did not lose their membrane integrity (PI negative) were included in the analysis for relative intracellular ion concentrations.

### Flow cytometric analysis of cytochrome c and glutathione

Analysis of cytochrome release from viable cells was determine by initially staining the cells with MitoTracker Deep Red (200 nM final) for 30 min at 37 °C, 7% CO_2_ atmosphere prior to harvest. The cells were washed once in 1X PBS, resuspended in 500 ul CytoFix (BD Biosciences) while vortexing, and held at room temperature for 30 min after which 1 ml of Perm Wash buffer (BD Biosciences) was added. The cells were centrifuged, resuspended in 100 ul of Perm Wash buffer, and stored overnight at 4 °C. The next day 2 ul of a 1:10 dilution of an Alexa Fluor 488 anti-cytochrome c antibody (BioLegend, Cat. No. 612304) and DAPI (50 ng/ml final) was added to each sample, incubated at room temperature for 60 min after which 500 ul of Perm Wash buffer was added. The cells were centrifuged, resuspended in 200 ul of Perm Wash buffer. Analysis of intracellular glutathione was determined by adding mBCl (10 uM final; Life Technologies) to each sample for 10 min at 37 °C, 7% CO_2_ atmosphere prior to examination. PI (10 ug/ml) as vital dye was added immediately prior to flow cytometric examination. All samples were analyzed using a Becton Dickinson LSRII equipped with FACSDiVa software for acquisition and analysis.

### RNA-seq data analysis method

S49 (Bcl-2) cells were treated with and without dexamethasone for 24 h or 48 h. Total RNA was extracted and purified from cell using a QIAGEN RNeasy RNA isolation kit (Redwood City, CA) according to the manufacturer’s protocol. RNA-seq libraries were generated with 1 mg of RNA as input using the TruSeq RNA Sample Prep Kit (Illumina, San Diego, CA) and poly(A)-enriched according to the TruSeq protocol. Indexed samples were sequenced using the 75 bp paired-end protocol via the NextSeq500 (Illumina) according to the manufacturer’s protocol. Reads (30–60 million reads per sample) were filtered based on a mean base quality score >20, and adapters were trimmed by “Trim Galore” (version 0.4). Trimmed reads were mapped to the University of California Santa Cruz (UCSC) mm10 reference genome using STAR(V 2.5.1). The quantification results from “featureCount (subread, Version 1.4.6)” were then analyzed with the Bioconductor package DESeq2, which fits a negative binomial distribution to estimate technical and biological variability. PCA plot was generated to identify sample outlier. Comparisons were made between different times of control and dexamethasone-treated cells. A gene was considered differentially expressed when the *p* value for differential expression was less than 0.01. Ingenuity pathway analysis (IPA) was performed on the differentially expressed genes to determine functional enrichment. The RNA-seq data has been deposited in the Gene Expression Omnibus with the accession number GSE210375.

### Statistics

One-way ANOVA followed by Tukey’s multiple comparison tests or paired *t* tests were used to evaluate the statistical relevance of control and experimental samples. A *p*-value of at least < 0.05 was considered significant.

## Supplementary information

Supplemental Figure Legends

Supplemental Figure 1

Supplemental Figure 2

Supplemental Figure 3

## Data Availability

All data are available in the main text or the supplementary materials.
