# Supplementary material for: Overcoming apoptotic resistance afforded by Bcl-2 in lymphoid tumor cells: a critical role for dexamethasone
Source: Cell Death Discov. 2022 Dec 20;8:494. doi: 10.1038/s41420-022-01285-x (PMC9767920; doi:10.1038/s41420-022-01285-x)
Supplement: Supplementary file 1 — Supplemental Figure Legends [file 41420_2022_1285_MOESM1_ESM.docx]

Supplementary Figure 1: Bcl-2 expression in S49 (Neo) and S49 (Bcl-2) cells. S49 (Neo) and S49 (Bcl-2) cells were fixed and permeabilized prior to staining with a PE-isotype or PE mouse anti-human Bcl-2 antibody and examined by flow cytometry. Only the S49 (Bcl-2) cells stained with the PE mouse anti-human Bcl-2 antibody showed an increase in fluorescence indicating only these cells expressed Bcl-2. Data represent 1 of 3 independent experiments.

Supplementary Figure 2: Changes in intracellular calcium in dex-treated S49 (Neo) and S49 (Bcl-2) cells. S49 (Neo) and S49 (Bcl-2) cells were treated with 2.5 x10^-7^ M dex for 48h and analyzed for changes in intracellular calcium using Fluo-4. Cells were examined on a Fluo-4 (calcium) vs PI fluorescence dot plot. No significant change in intracellular calcium was observed under all conditions. Data represent 1 of 3 independent experiments.

Supplementary Figure 3: Microbial toxins that override apoptotic resistance in Bcl-2 expressing cells show an increase in intracellular calcium. S49 (Bcl-2) cells were treated with 2.5 x10^-7^ M dex for 48h in the presence or absence of 25 nM valinomycin, 25 nM cereulide, or 10 uM salinomycin during the final 6 hours and examined for changes in intracellular calcium using Fluo-4. Cells were examined on a Fluo-4 (calcium) vs PI fluorescence dot plot. Data represent 1 of 3 independent experiments.
