# Supplementary figures and images for: Overcoming apoptotic resistance afforded by Bcl-2 in lymphoid tumor cells: a critical role for dexamethasone
Source: Cell Death Discov. 2022 Dec 20;8:494. doi: 10.1038/s41420-022-01285-x (PMC9767920; doi:10.1038/s41420-022-01285-x)

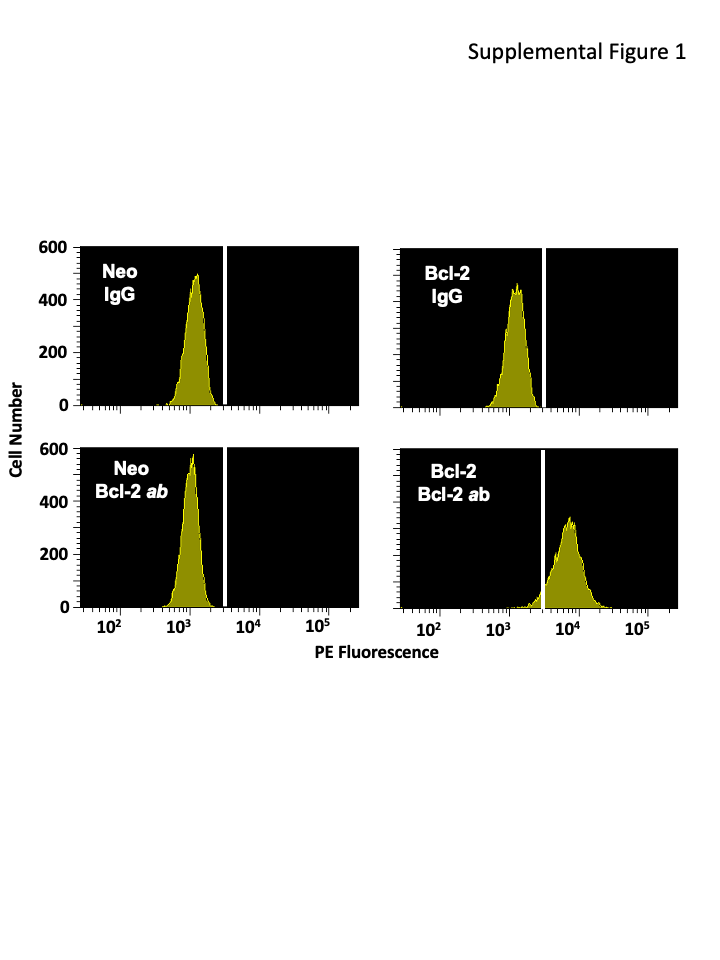

Supplement: Supplementary file 2 — Supplemental Figure 1 [file 41420_2022_1285_MOESM2_ESM.tif]

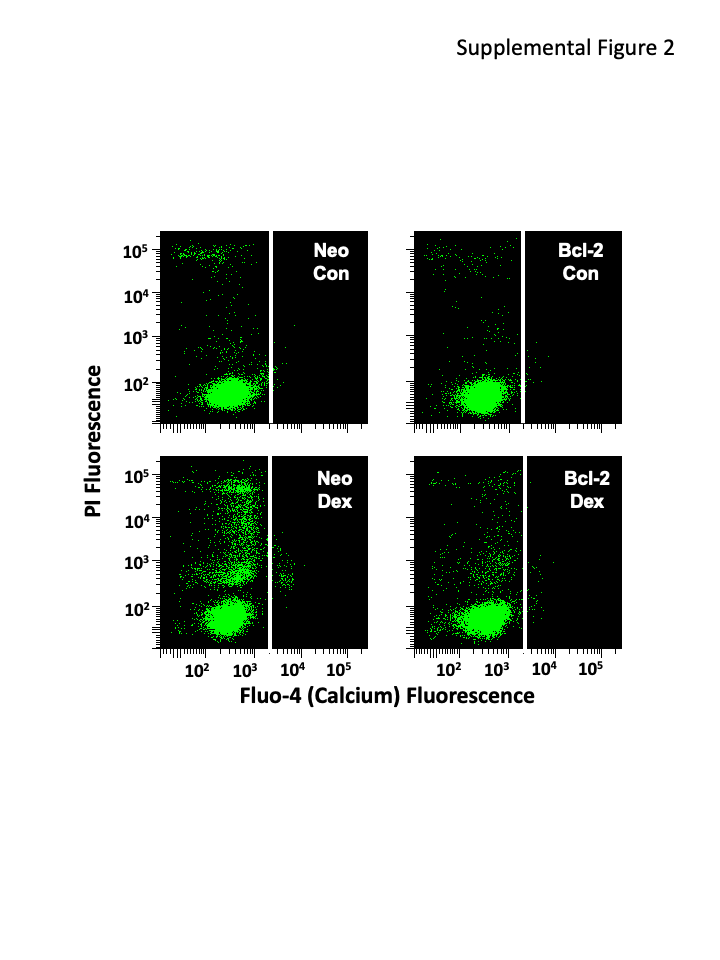

Supplement: Supplementary file 3 — Supplemental Figure 2 [file 41420_2022_1285_MOESM3_ESM.tif]

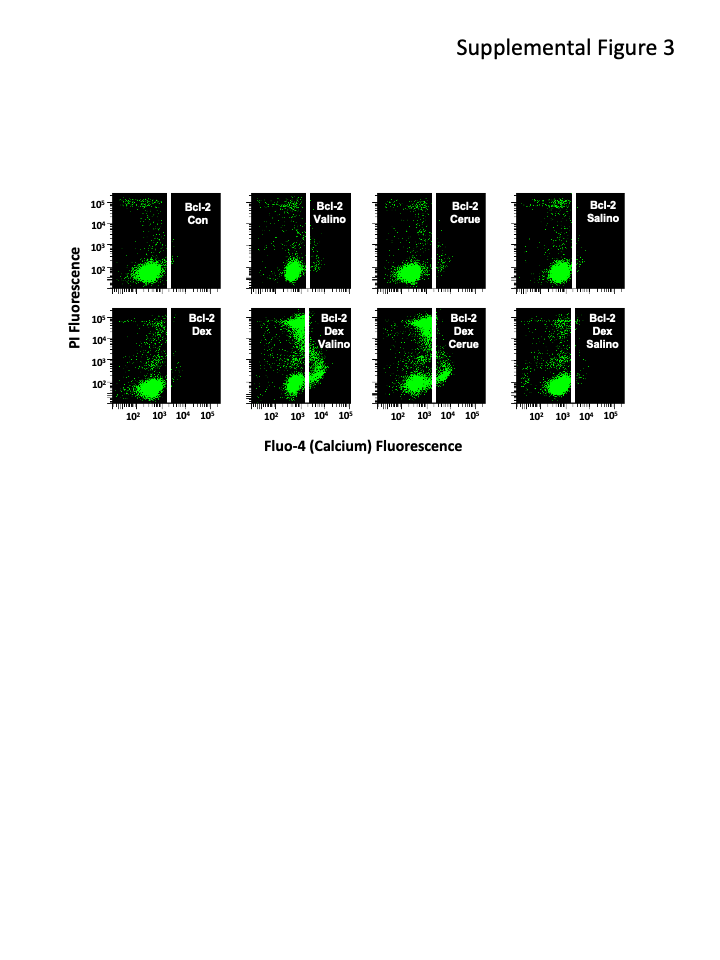

Supplement: Supplementary file 4 — Supplemental Figure 3 [file 41420_2022_1285_MOESM4_ESM.tif]
